# SARS-CoV-2 Causes Acute Kidney Injury by Directly Infecting Renal Tubules

**DOI:** 10.3389/fcell.2021.664868

**Published:** 2021-05-31

**Authors:** Zhaohui Chen, Junyi Hu, Lilong Liu, Rong Chen, Miao Wang, Ming Xiong, Zhen-Qiong Li, Yi Zhao, Hong Li, Chuhuai Guan, Jie Zhang, Liang Liu, Ke Chen, Yu-Mei Wang

**Affiliations:** ^1^Department of Urology, Union Hospital, Tongji Medical College, Huazhong University of Science and Technology, Wuhan, China; ^2^Department of Pathology, Jin Yin-tan Hospital, Wuhan, China; ^3^Department of Nephrology, Union Hospital, Tongji Medical College, Huazhong University of Science and Technology, Wuhan, China; ^4^Department of Ultrasound, Union Hospital, Tongji Medical College, Huazhong University of Science and Technology, Wuhan, China; ^5^Department of Forensic Medicine, Tongji Medical College of Huazhong University of Science and Technology, Wuhan, China

**Keywords:** COVID-19, SARS-CoV-2, AKI, ACE2, TMPRSS2, RNAscope

## Abstract

Acute kidney injury (AKI) is one of the most prevalent complications among hospitalized coronavirus disease 2019 (COVID-19) patients. Here, we aim to investigate the causes, risk factors, and outcomes of AKI in COVID-19 patients. We found that angiotensin-converting enzyme II (ACE2) and transmembrane protease serine 2 (TMPRSS2) were mainly expressed by different cell types in the human kidney. However, in autopsy kidney samples, severe acute respiratory syndrome coronavirus 2 (SARS-CoV-2) nucleoprotein was detected in ACE2^+^ or TMPRSS2^+^ renal tubular cells, whereas the RNAscope^®^ Assay targeting the SARS-CoV-2 Spike gene was positive mainly in the distal tubular cells and seldom in the proximal tubular cells. In addition, the TMPRSS2 and kidney injury marker protein levels were significantly higher in the SARS-CoV-2-infected renal distal tubular cells, indicating that SARS-CoV-2-mediated AKI mainly occurred in the renal distal tubular cells. Subsequently, a cohort analysis of 722 patients with COVID-19 demonstrated that AKI was significantly related to more serious disease stages and poor prognosis of COVID-19 patients. The progressive increase of blood urea nitrogen (BUN) level during the course of COVID-19 suggests that the patient’s condition is aggravated. These results will greatly increase the current understanding of SARS-CoV-2 infection.

## Introduction

The outbreak of coronavirus disease 2019 (COVID-19) has evolved into a global pandemic. Until October 10, 2020, there were over 36 million confirmed cases, and 1,056,186 deaths have been reported (WHO, 2019). Among COVID-19 patients, acute kidney injury (AKI) is one of the most prevalent complications (36.6%) and is associated with a poor prognosis (Cheng et al., 2020b; Hirsch et al., 2020). However, it is still not clear that AKI caused by COVID-19 was either directly a damage of infection or a manifestation of systemic inflammatory response. Severe acute respiratory syndrome coronavirus 2 (SARS-CoV-2) was the pathogen of the COVID-19 pandemic. Cell entry of SARS-CoV-2 depends on binding of the viral spike (S) proteins to cellular receptors and on S protein priming by host cell proteases. Previous studies (Hoffmann et al., 2020; Zheng et al., 2020) have demonstrated that SARS-CoV-2 uses the SARS-CoV receptor angiotensin-converting enzyme II (ACE2) for entry and the transmembrane protease serine 2 (TMPRSS2) for S protein priming. In addition, ACE2 has been found to be highly expressed in renal tubules (Fu et al., 2020) and upregulated in patients with COVID-19, and immunostaining with SARS-CoV nucleoprotein (NP) antibody was positive in tubules (Su et al., 2020b). SARS-CoV-2 RNA was also detected in the urine sample of COVID-19 patients, and the positive urine duration was more than 1 month (Sun et al., 2020). Notably, Sun et al. (2020) successfully isolated infectious SARS-CoV-2 from the urine of a COVID-19 patient. Collectively, these data indicated that SARS-CoV-2 might be secreted through the human urinary system and tubules could potentially be infected by SARS-CoV-2.

In this study, we found that ACE2 and TMPRSS2 were mainly expressed by different cell types in the human kidney. Interestingly, SARS-CoV-2 nucleoprotein was detected in ACE2^+^ or TMPRSS2^+^ renal tubular cells, whereas the RNAscope^®^ Assay targeting the SARS-CoV-2 Spike gene was positive mainly in the distal tubular cells and seldom in the proximal tubular cells. These results indicate that the kidney is a direct target for SARS-CoV-2 infection; however, the way how SARS-CoV-2 enters the renal tubular cells was still unknown. Furthermore, we found that TMPRSS2 and kidney injury makers were upregulated in COVID-19 patients’ renal tubular cells. Subsequently, a cohort analysis of 722 patients with COVID-19 demonstrated that AKI was significantly related to more serious disease stages and poor prognosis of COVID-19 patients. The progressive increase of blood urea nitrogen (BUN) level during the course of COVID-19 suggests that the patient’s condition is aggravated. These results will greatly increase the current understanding of SARS-CoV-2 infection.

## Results

### Histological Location of ACE2 and SARS-CoV-2 in Human Kidney

**FIGURE 1:**
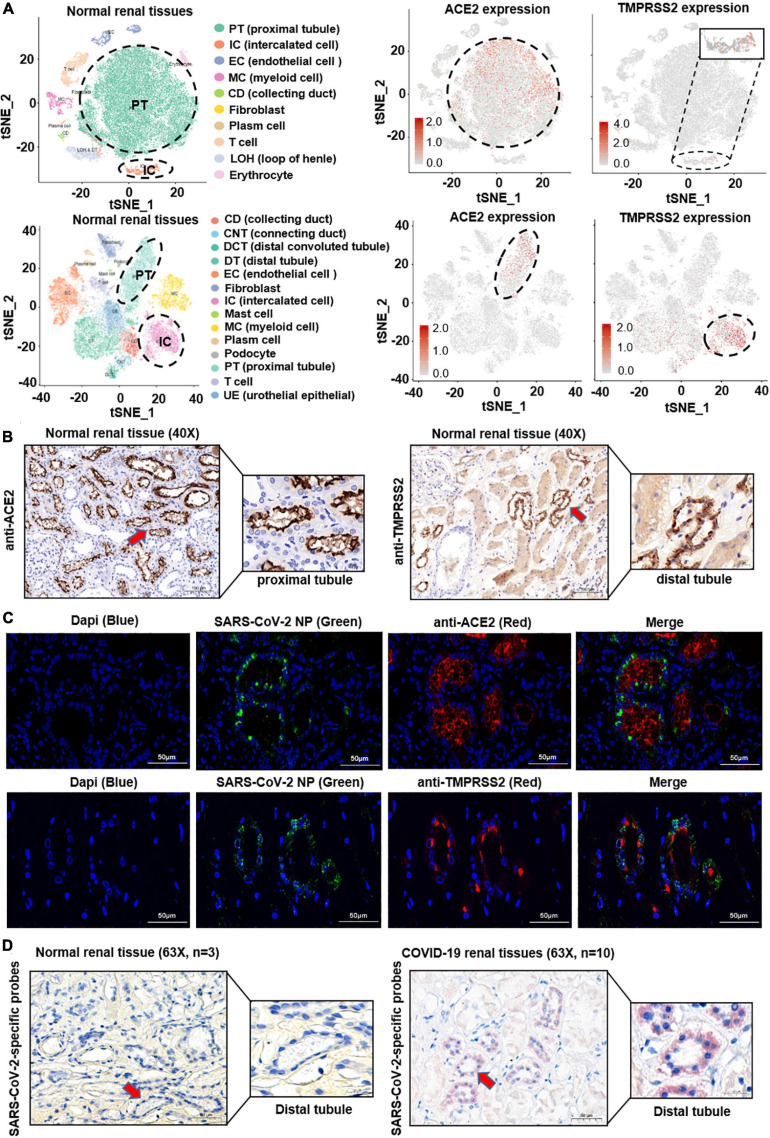
Histological location of ACE2 and SARS-CoV-2 in the human kidney. **(A)** The public single-cell RNA sequencing datasets based on two different platforms (10X Genomics and Microwell-seq) were analyzed. After quality control, dimension descending, and cell type identification, major cell types in the kidney were shown in the t-SNE plot. ACE2 was highly expressed in the proximal tubular cells, whereas TMPRSS2 was highly expressed in the intercalated cells. **(B)** ACE2 and TMPRSS2 protein levels were analyzed in normal kidney tissues (n = 3). ACE2 signature was detected mainly on the apical side of the proximal tubular cells. In contrast, TMPRSS2 was mainly localized at the distal tubular cells. **(C)** SARS-CoV-2 nucleoprotein was co-stained with ACE2 or TMPRSS2 by Immunofluorescence (IFC) staining in 10 COVID-19 patients’ kidney samples. SARS-CoV-2 nucleoprotein was detected in ACE2^+^ or TMPRSS2^+^ renal tubular cells. **(D)** A novel *in situ* hybridization assay (RNAscope^®^ Assay) targeting the SARS-CoV-2 Spike gene was positive in the kidney distal tubular cells of COVID-19 patients.

Severe acute respiratory syndrome coronavirus 2 enters human cells through SARS-CoV receptor ACE2 and shows in a TMPRSS2-dependent manner (Hoffmann et al., 2020; Zheng et al., 2020). In order to investigate the localization of both the ACE2 and TMPRSS2 in the human kidney, we firstly performed bioinformatic analysis on public single-cell RNA sequencing (scRNA-seq) datasets based on two different platforms (10X Genomics and Microwell-seq). After quality control, dimension descending, and cell type identification (see the Methods section), major cell types in the kidney were shown in the T-Distributed Stochastic Neighbor Embedding (t-SNE) plot. As shown in Figure 1A and Supplementary Figures 1A,B, ACE2 was highly expressed in the proximal tubular cells and hardly expressed in other cells, whereas TMPRSS2 was highly expressed in the intercalated cells and moderately expressed in the distal tubule, collecting duct, collecting system, distal convoluted tubule, and urothelial epithelial, but less expressed in the proximal tubule.

Next, in order to validate this finding, we performed immunohistochemistry (IHC) staining assay on normal kidney samples. As shown in Figure 1B, ACE2 signature was detected mainly on the apical side of the proximal tubular cells. Instead, TMPRSS2 was mainly localized at the distal tubular cells, whereas at the proximal tubule in a relatively low level. These findings corresponded to the results of scRNA-seq analysis.

The difference of histological localization of ACE2 and TMPRSS2 in the human kidney has led to our attention. Next, to investigate the localization of SARS-CoV-2 in the human kidney, SARS-CoV-2 nucleoprotein was co-stained with ACE2 or TMPRSS2 by Immunofluorescence (IFC) staining in 10 COVID-19-infected kidney samples, respectively. As shown in Figure 1C and Supplementary Figures 2A,B, SARS-CoV-2 nucleoprotein was detected in ACE2^+^ or TMPRSS2^+^ renal tubular cells. Furthermore, we used a novel *in situ* hybridization assay (RNAscope^®^ Assay) to detect SARS−CoV−2 RNA in fixed kidney specimen sections. Intense signals were observed mainly in the distal tubular cells and seldom in the proximal tubular cells of COVID-19 patient’s kidney tissues (Figure 1D). These results indicated that the renal distal tubule is a direct target for SARS-CoV-2 infection; however, SARS-CoV-2 may enter the distal tubular cells in an ACE2-independent manner, and which protein served as the receptor for SARS-CoV-2 on TMPRSS2^+^ cells is unknown.

### SARS-CoV-2 Infection Causes Direct Damage in the Kidney

**FIGURE 2:**
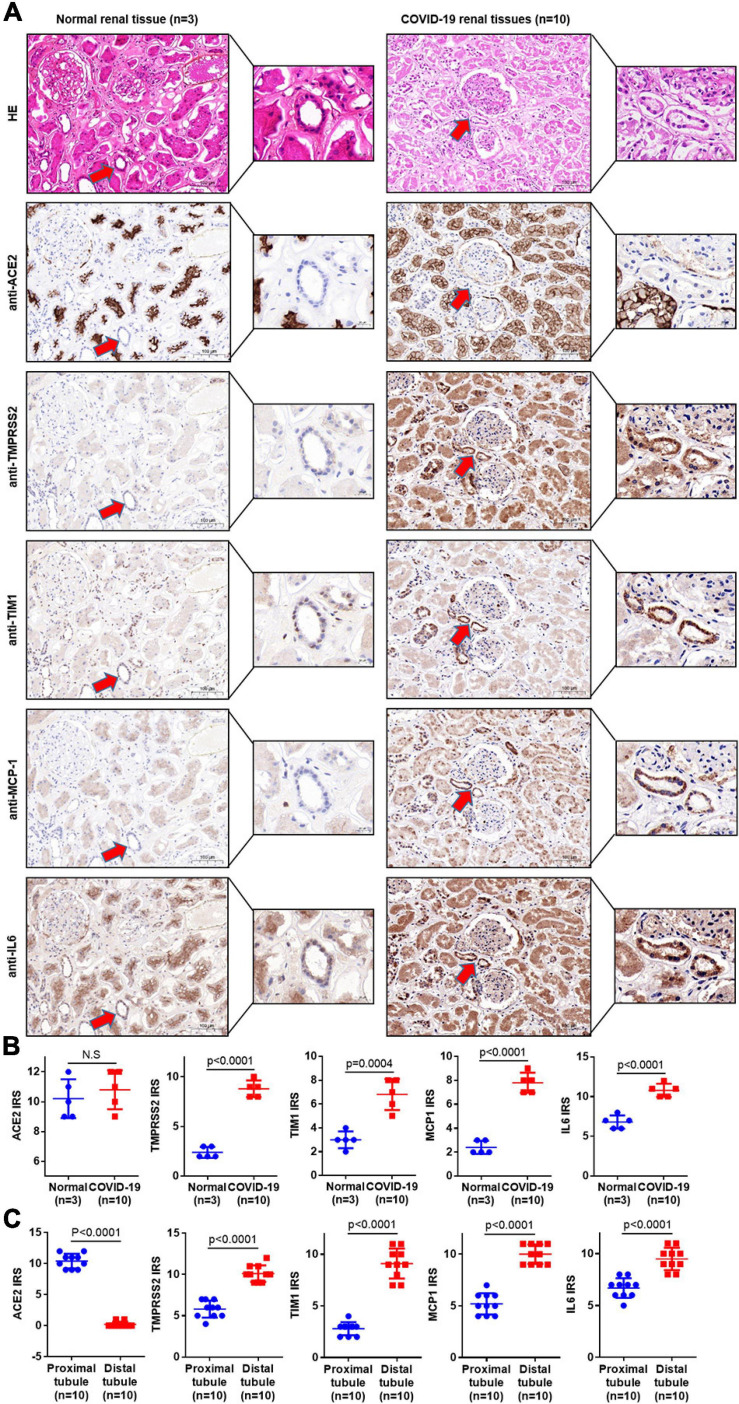
COVID-19 infection causes direct damage in the kidney. **(A)** Serial sections (5 μm thick) were prepared for H&E staining and IHC staining. H&E staining was used to distinguish the proximal tubules from the distal tubules. IHC staining was used to detect the related molecules of classical kidney injury in 10 COVID-19 patients’ kidney samples and 3 normal kidney samples. **(B)** The immunoreactive score (IRS) measured the expression of ACE2, TMPRSS2, TIM1, IL-6, and MCP-1 in normal and COVID-19 patients’ kidney samples. **(C)** The IRS measured the expression of ACE2, TMPRSS2, TIM1, IL-6, and MCP-1 in the distal tubule and proximal tubule of COVID-19 patients. Bar graphs show mean ± SD, Student’s *t*-test, *p* < 0.05 was statistically significant; NS, no significance.

**FIGURE 3:**
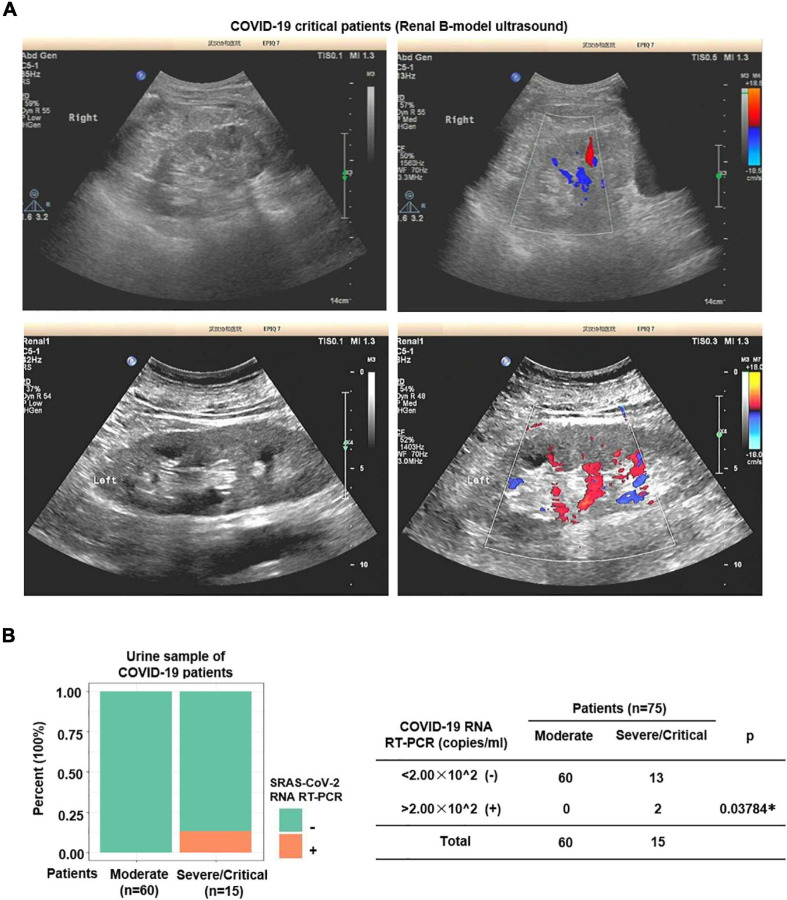
Nucleic acid of SARS-CoV-2 was detected in urine of severe patients. **(A)** Color Doppler imaging showed reduced perfusion in severe COVID-19 patients. **(B)** SARS-CoV-2 RNA Real-Time PCR was performed on urine samples. SARS-CoV-2 nucleic acid was detected in 2 out of 15 (13.3%) severe samples, whereas none of the moderate samples (*n* = 60) was positive.

In order to further investigate the influence of direct infection of SARS-CoV-2 in the human kidneys, we performed hematoxylin–eosin (H&E) staining and IHC staining of classical kidney injury-related molecules on paraffin samples serial sections (5 μm thick). As shown in Figure 2, TMPRSS2 was upregulated in the distal tubule of all the severe COVID-19 patients’ kidney samples. Interestingly, T cell Ig and mucin domain-containing protein-1 (TIM-1), also known as kidney injury molecule-1 (KIM-1), was highly expressed in these TMPRSS2^+^ cells, whereas rarely expressed in the proximal tubules. In previous studies, TIM-1 was reported to be upregulated in the proximal tubules in AKI patients (Ajay et al., 2014; Tajima et al., 2019). It indicated that SARS-CoV-2-mediated kidney injury may mainly target the distal tubular cells. Additionally, inflammation-related molecules, including MCP-1 and interleukin 6 (IL-6), were enriched in severe COVID-19 kidneys, especially in the distal tubules (Figure 2). In addition, Color Doppler imaging showed reduced perfusion, and the resistance index (RI) of the intrarenal interlobular/interlobular artery increased in severe COVID-19 patients (Figure 3A). All the results indicated that the kidney is a direct target for SARS-CoV-2 infection and the damage occurred mainly in the distal tubule.

### SARS-CoV-2 RNA Is Detected in Severe Patients’ Urine

The renal distal tubule was highly damaged in severe COVID-19 patients. A previous study has shown that SARS-CoV-2 can be excreted through the urinary system (Sun et al., 2020). Next, to further investigate whether SARS-CoV-2 could get into the urine, we performed SARS-CoV-2 RNA Real-Time PCR on urine samples. As shown in Figure 3B, SARS-CoV-2 RNA was detected in 2 out of 15 (13.3%) severe samples, whereas none of the moderate samples was positive.

### Occurrence of AKI Indicates Poor Prognosis in COVID-19 Patients

To further understand the characteristics of COVID-19 patients, we investigated a cohort consisting of 722 COVID-19 patients treated at the West Branch of Union Hospital of Huazhong University of Science and Technology between January 26, 2020 and March 31, 2020 (Tables 1, 2). Compared with female patients, male patients are more likely to have critical disease. Critical patients are significantly older than moderate and severe patients. Over half of these patients have comorbidities (435/722), with hypertension ranked at the first, followed by diabetes and cancer. In this cohort, 9 (1.86%) out of 484 severe patients and 50 (26.3%) out of 190 critical patients underwent AKI, whereas none of the moderate group showed this complication. Patients with more serious disease show significantly higher possibility to suffer from AKI (Tables 1, 2). Moreover, 45 (77.6%) out of 58 patients with AKI died in the end [odds ratio (OR) = 20.67, 95% confidence interval (CI), 10.53–43.22, *p* < 0.0001], revealing that AKI indicates poor prognosis of COVID-19 patients.

**TABLE 1:** Demographic information of COVID-19 patients.

		**Moderate (*n* = 38)**	**Severe (*n* = 494)**	**Critical (*n* = 190)**		**Moderate vs severe**	**Moderate vs critical**	**Severe vs critical**
Death		0 (0%)	0 (0%)	145 (76.3%)	<0.0001	1	<0.0001	<0.0001
Gender	Male	21 (55.3%)	236 (47.8%)	127 (66.8%)				
	Female	17 (44.7%)	258 (52.2%)	63 (33.2%)	<0.0001	0.4031	0.2915	<0.0001
Age	≤60	26	273	59				
	>60	12	221	131	<0.0001	0.1288	<0.0001	<0.0001
Ventilation	Non-Invasive	0 (0%)	5 (1%)	110 (57.9%)	<0.0001	1	<0.0001	<0.0001
	Invasive	0 (0%)	3 (0.6%)	75 (39.5%)	<0.0001	1	<0.0001	<0.0001
**Complication**								
ARDS		0 (0%)	44 (8.9%)	184 (96.8%)	<0.0001	0.062	<0.0001	<0.0001
DIC		0 (0%)	3 (0.6%)	64 (33.7%)	<0.0001	1	<0.0001	<0.0001
Shock		0 (0%)	0 (0%)	145 (76.3%)	<0.0001	1	<0.0001	<0.0001
AKI		0 (0%)	8 (1.6%)	50 (26.3%)	<0.0001	1	<0.0001	<0.0001
Liver injury		15 (39.5%)	303 (61.3%)	153 (80.5%)	<0.0001	0.0098	<0.0001	<0.0001
Myocardial injury		3 (7.9%)	63 (12.8%)	102 (53.7%)	<0.0001	0.6074	<0.0001	<0.0001
Thrombocytopenia		1 (2.6%)	22 (4.5%)	88 (46.3%)	<0.0001	1	<0.0001	<0.0001
**Comorbidities**								
Total		19 (50%)	282 (57.1%)	135 (71.1%)	0.0015	0.4017	0.0221	0.0026
Cancer		2	24	18	0.0759			
Diabetes		5	81	37	0.5102			
Hypertension		7	145	74	0.0121	0.1922	0.016	0.0175
**Symptom**								
Fever		26 (68.4%)	363 (73.5%)	150 (78.9%)	0.2243			
Cough		19 (50%)	345 (69.8%)	137 (72.1%)	0.0243	0.026	0.026	0.5761
Dyspnea		8 (21.1%)	176 (35.6%)	115 (60.5%)	<0.0001	0.0775	<0.0001	<0.0001
Fatigue		18 (47.4%)	249 (50.4%)	121 (63.7%)	0.0055	0.7394	0.1043	0.0061
Myalgia		8 (21.1%)	120 (24.3%)	45 (23.7%)	0.8985			
Diarrhea		2 (5.3%)	86 (17.4%)	29 (15.3%)	0.1352			

**TABLE 2:** Laboratory information of COVID-19 patients on admission.

	**Moderate (*n* = 38)**	**Severe (*n* = 494)**	**Critical (*n* = 190)**		**Moderate vs severe**	**Moderate vs critical**	**Severe vs critical**
White blood cell count, 10^9^/L	5.3 (4.47–6.18)	5.58 (4.43–7.1)	8.14 (5.86–10.9)	<0.0001	0.9744	<0.0001	<0.0001
Hemoglobin, g/dl	129 (119–137.75)	125 (115–136)	129 (117–140)	0.0901			
Platelet count, 10^9^/L	222.5 (153.75–254.75)	219 (167–280)	174.5 (121.25–235.75)	<0.0001	1	0.0595	<0.0001
Neutrophil count, 10^9^/L	3.48 (2.72–3.95)	3.73 (2.81–5.19)	7.08 (4.53–9.94)	<0.0001	0.3757	<0.0001	<0.0001
Lymphocyte, 10^9^/L	1.30 (0.83–1.65)	1.11 (0.82–1.57)	0.6 (0.4–0.86)	<0.0001	0.8203	<0.0001	<0.0001
**Proteinuria**							
+	0	20	36				
-	17	202	40	<0.0001	0.3736	0.0002	<0.0001
**BLD-U**							
+	2	31	27				
-	15	189	49	0.0002	1	0.0812	0.0002
**U-WBC**							
+	3	31	9				
-	14	190	65	0.8048			
D-dimer, mg/L	0.33 (0.2–0.76)	0.48 (0.25–1.12)	4.16 (0.76–8)	<0.0001	0.3189	<0.0001	<0.0001
PT, s	12.8 (12.55–13.35)	13.1 (12.4–13.8)	14.2 (13.2–15.2)	<0.0001	1	<0.0001	<0.0001
APTT, s	37.9 (35.3–41.6)	36.2 (33.1–39.7)	38.2 (32.9–44.2)	0.0008	0.1397	1	0.0019
Total protein, g/L	64.1 (60.4–67.15)	63 (59.5–66.93)	61.4 (58.1–65.35)	0.012585	1	0.1268	0.0192
ALB, g/L	34.9 (31.15–38.83)	32.2 (28.78–36.6)	27.9 (25.25–31.28)	<0.0001	0.0301	<0.0001	<0.0001
AST, U/L	25 (18–32)	28 (21–41)	45 (31–63.5)	<0.0001	0.221	<0.0001	<0.0001
ALT, U/L	22.5 (19–44)	32 (20–51)	39.5 (25–64)	0.0003	0.5338	0.0079	0.001
LDH, U/L	202 (152.25–249)	231 (184–310)	506 (356.5–635.5)	<0.0001	0.0779	<0.0001	<0.0001
CK, U/L	55.5 (40.75–103.75)	70 (46–123.75)	126 (64.5–282)	<0.0001	0.5234	1.00E-04	<0.0001
BUN, mmol/L	4.22 (3.37–5.18)	4.47 (3.41–5.8)	6.62 (5.09–9.61)	<0.0001	0.9760	<0.0001	<0.0001
Scr, μmol/L	69.05 (58.05–80.28)	65.1 (55.8–78.03)	74 (62.58–90.12)	<0.0001	0.9071	0.5359	<0.0001
C-reactive protein, mg/L	7.36 (2.46–29.1)	15.28 (3.38–47.44)	80.99 (41.87–123.46)	<0.0001	0.9496	<0.0001	<0.0001
PCT, ng/ML	0.06 (0.04–0.1)	0.07 (0.05–0.12)	0.27 (0.13–0.63)	<0.0001	0.8327	<0.0001	<0.0001
ESR, mm/h	28 (21–47)	39 (24–65.5)	59.5 (33.5–75)	0.0073	1	0.1241	0.0109
Serum ferritin, ng/ml	714.55 (256.54–1,081.38)	473.51 (205.25–765.09)	1,206.36 (531.02–2,000)	<0.0001	1	0.2903	<0.0001

### The Progressive Increase of BUN Level Indicates Aggravation of the COVID-19 Disease

Next, to further investigate potential risk factors and monitoring indicators of AKI, we re-examined all the variables between AKI and no AKI cases. Since AKI occurs only in severe and critical cases, moderate COVID-19 patients were not involved in this investigation. Additionally, in order to eliminate the deviation caused by comorbidities, 21 patients with chronic kidney diseases were excluded. As shown in Table 3, the median age of the no AKI group was 60 (IQR, 50–67), whereas the AKI group was significantly older (median, 69; IQR, 63–74.75). Compared with female patients, male patients show higher potential to emerge from AKI. Whether in the AKI or no AKI group, over half of the patients have comorbidities.

**TABLE 3:** Laboratory findings between COVID-19 patients with AKI and without AKI on admission.

	**No AKI (*n* = 605)**	**AKI (*n* = 58)**	
Age, years	60 (50–67)	69 (63–74.75)	<0.0001
Gender	–	–	–
Male	307	45	–
Female	298	13	0.0002
Comorbidities	350	46	0.0023
**Symptom**			
Fever	429 (75%)	48 (83%)	0.2401
Cough	429 (71%)	40 (69%)	0.8731
Dyspnea	245 (40%)	33 (57%)	0.0240
Fatigue	325 (54%)	36 (62%)	0.2794
Myalgia	144 (24%)	15 (26%)	0.8492
Diarrhea	104 (17%)	7 (12%)	0.4158
White blood cell count, 10^9^/L	5.88 (4.48–7.58)	8.73 (6.67–10.99)	<0.0001
Hemoglobin, g/dl	126 (116–137)	130 (118.25–143)	0.1001
Platelet count, 10^9^/L	214.5 (160–276.25)	171 (122.75–222.75)	0.0001
Neutrophil count, 10^9^/L	3.95 (2.88–5.99)	7.3 (5.55–10.24)	<0.0001
Lymphocyte, 10^9^/L	1.03 (0.7–1.48)	0.58 (0.39–0.92)	<0.0001
D-dimer, mg/L	0.58 (0.28–2.09)	3.74 (0.71–8)	<0.0001
PT, s	13.2 (12.5–14.1)	14.1 (13–15.53)	0.0001
APTT, s	36.4 (33–40.58)	36.95 (33.53–41.83)	0.2086
Total protein, g/L	62.7 (59.2–66.55)	62.35 (58.98–66.83)	0.9037
ALB, g/L	31.4 (27.9–35.6)	28.05 (25.1–32.03)	<0.0001
AST, U/L	31 (22–45)	48.5 (31–66.75)	<0.0001
ALT, U/L	33 (21–54.5)	37 (29.25–64)	0.0242
LDH, U/L	256 (194–364)	534.5 (400.25–669)	<0.0001
CK, U/L	74 (47–148)	144.5 (65.5–297.5)	<0.0001
BUN, mmol/L	4.66 (3.62–6.15)	8.41 (5.99–11.75)	<0.0001
Scr, μmol/l	66.2 (56–77.4)	85.75 (69.65–136.38)	<0.0001
C-reactive protein, mg/L	23.21 (4.16–64.97)	92.29 (40.75–124.52)	<0.0001
PCT, ng/ml	0.08 (0.05–0.15)	0.31 (0.15–0.9)	<0.0001
ESR, mm/h	42 (25–66)	65 (33–75)	0.1204
Serum ferritin, ng/ml	498.2 (248.97–857.8)	1,347.53 (1,033.83–2,000)	<0.0001
Proteinuria	–	–	–
+	17	36	–
-	13	225	<0.0001
BLD-U	–	–	–
+	12	43	–
-	18	216	0.0044
U-WBC	–	–	–
+	6	33	–
-	24	225	0.4177

When comparing laboratory indexes between severe COVID-19 patients with or without AKI, we found that white blood cell (WBC) and neutrophil counts were higher in AKI patients, whereas lymphocyte and platelet counts were lower. In biochemical profile, AKI patients show higher levels of D-dimer, aspartate aminotransferase (AST), alanine aminotransferase (ALT), lactic dehydrogenase (LDH), creatine kinase (CK), C-reactive protein (CRP), procalcitonin (PCT), and serum ferritin, whereas albumin (ALB) significantly decreased in AKI patient. Notably, BUN and serum creatinine (Scr) levels are also higher in AKI patients (Table 3). These results indicated that AKI patients show greater evidence of multi-organ damage than the no AKI group.

**FIGURE 4:**
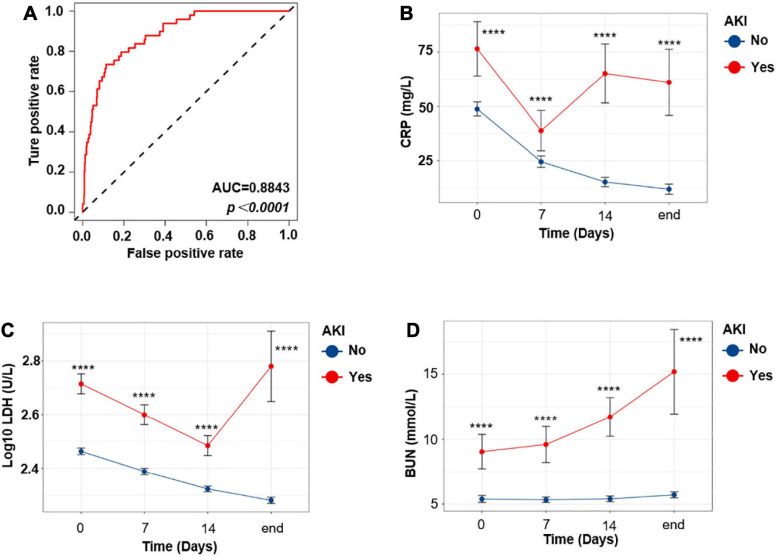
The progressive increase of BUN level indicates aggravation of the COVID-19 disease. **(A)** ROC curve of the multivariate model. **(B–D)** Temporal change in CRP **(B)**, LDH **(C)**, and BUN **(D)**. Differences between the AKI and no AKI groups were significant for all timepoints shown. Bar represents mean ± SEM. *p*-Value was calculated by Mann–Whitney U test. ****p < 0.0001.

When exploring risk factors of AKI in COVID-19 patients, we performed receiver operating characteristic (ROC) analysis and univariate logistic regression at first. Only variables with area under the curve (AUC) >0.6 and show statistical significance in univariate logistic regression model were included in the multivariate analysis (detailed in the section “Materials and Methods”). Finally, only six variables were included in this model. Hosmer and Lemeshow test was conducted to confirm the goodness of fit of this model (Pr = 0.6554 > 0.1). The ROC curve of this model is shown in Figure 4A (AUC = 0.8842). As shown in Table 4, in this multivariate analysis, LDH, BUN, and CRP were associated with increased risk of AKI, in which BUN shows the highest OR (1.12, 95% CI, 1.04–1.20). In order to further the diagnostic value of these indexes, we investigated these variables of COVID-19 patients on admission, 7 and 14 days after admission, and at the end of the follow-up, respectively (Figures 4B–D). Interestingly, although LDH and CRP levels were higher in the AKI group at any time point, they decreased at the beginning of the treatment (Figures 4B,C). However, in the AKI group, BUN level showed a continuous increase during hospitalization (Figure 4D); this suggests that BUN is progressive with the aggravation of the disease.

**TABLE 4:** Factors associated with AKI of COVID-19 patients.

	**AUC**	**Univariable logistic regression**		**Multivariable logistic regression**	
		**OR (95% CI)**	***p*-Value**	**OR (95% CI)**	***p*-Value**
Age	0.7142	1.06 (1.03, 1.08)	<0.001*	1.03 (0.99, 1.06)	0.109
Gender	–	3.36 (1.78, 6.36)	<0.001*	2.22 (0.96, 5.12)	0.062
Morbidities	–	2.79 (1.45, 5.38)	0.002*	1.95 (0.83, 4.61)	0.128
White blood cell count, 10^9^/L	0.7255	1.05 (1, 1.11)	0.068	–	–
Hemoglobin, g/dl	0.5653	0.16 (0.08, 0.33)	<0.001	–	–
Platelet count, 10^9^/L	0.6518	1.18 (1.12, 1.25)	<0.001*	–	–
Neutrophil count, 10^9^/L	0.7632	0.9935 (0.99, 0.997)	<0.001*	–	–
Lymphocyte, 10^9^/L	0.7281	1.27 (1.17, 1.39)	<0.001*	–	–
Proteinuria	–	8.17 (3.66, 18.25)	<0.001	–	–
BLD-U	–	3.35 (1.5, 7.46)	0.003	–	–
U-WBC	–	1.7 (0.65, 4.48)	0.279	–	–
D-dimer, mg/L	0.7186	1.27 (1.17, 1.39)	<0.001*	–	–
PT, s	0.6546	0.9996 (0.9942, 1.0049)	0.874	–	–
APTT, s	0.5509	1.04 (1, 1.07)	0.029	–	–
Total protein, g/L	0.4965	1.02 (0.98, 1.07)	0.279	–	–
ALB, g/L	0.6753	0.88 (0.83, 0.93)	<0.001*	–	–
AST, U/L	0.7132	1.0017 (1.0002, 1.0033)	0.031*	–	–
ALT, U/L	0.5896	1.0021 (1.0003, 1.004)	0.024	–	–
LDH, U/L	0.8303	1.0025 (1.0014, 1.0036)	<0.001*	1.0033 (1.0017, 1.0049)	<0.001
CK, U/L	0.6691	1.0014 (1.0007, 1.0021)	<0.001*	–	–
BUN, mmol/L	0.7882	1.22 (1.15, 1.3)	<0.001*	1.12 (1.04, 1.2)	0.003
Scr, μmol/l	0.7315	1.0062 (1.0014, 1.0111)	0.011*	–	–
C-reactive protein, mg/L	0.7695	1.02 (1.01, 1.02)	<0.001*	1.01 (1, 1.02)	<0.001
PCT, ng/ml	0.8294	1.3 (1, 1.67)	0.048*	–	–
ESR, mm/h	0.6134	1.01 (1, 1.03)	0.155	–	–
Serum ferritin, ng/ml	0.8205	1.0018 (1.0011, 1.0024)	<0.001	–	–

*P < 0.05

## Discussion

Although the respiratory and immune systems are the major targets of SARS-CoV-2, AKI and proteinuria have also been observed (Kudose et al., 2020; Su et al., 2020b). Previous studies have demonstrated that the kidney tissues from six postmortem cases have severe acute tubular necrosis but no evidence of glomerular pathology or tubulointerstitial lymphocyte infiltration by H&E staining (Diao et al., 2020), coupled with IHC showing that the SARS-CoV-2 nucleoprotein antigen was accumulated in the kidney tubules (Diao et al., 2020; Su et al., 2020b), suggesting that the SARS-CoV-2 viruses can directly infect the human kidney. In addition, electron microscopic examination showed clusters of coronavirus-like particles with distinctive spikes in the tubular epithelium and podocytes (Su et al., 2020b; Xia et al., 2020), highlighting the invasion of SARS-CoV-2 into the kidney tissue. However, another study (Sharma et al., 2020) revealed that ultrastructural examination by electron microscopy showed no evidence of viral particles in the biopsy samples of 10 hospitalized COVID-19 patients with AKI (acute tubular necrosis). Miller and Brealey (2020) purported that those structures of virus particles in the cytoplasm of the kidney tubular epithelium and podocytes as Su et al. (2020b) showed are not viral particles, but rather clathrin-coated vesicles, normal cell organelles involved in intracellular transport. Furthermore, Su et al. (2020a) stated that there are inherent difficulties in the discrimination of cellular vesicles from viral particles solely by morphological evidence, especially in routine electron microscopic examination processing of autopsy tissues. In contrast, *in situ* hybridization studies to assess local protein or RNA levels of CoV will further clarify the possibility of direct kidney parenchymal infection (Su et al., 2020a). Thus, there is no clear evidence that SARS-CoV-2 can directly infect the renal tubular cells.

The latest studies (Hoffmann et al., 2020; Zheng et al., 2020) have demonstrated that host cell entry of SARS-CoV-2 depends on the presence of ACE2 and TMPRSS2. Previous studies reported that ACE2 was highly expressed by the renal tubular cells (Fu et al., 2020; Su et al., 2020b), which suggested that these cells could potentially be infected by SARS-CoV-2. In the present study, we found that ACE2 and TMPRSS2 were expressed by different cell types in the human kidney. ACE2 signature was detected mainly on the apical side of the renal proximal tubular cells. TMPRSS2 was mainly localized at the renal distal tubular cells. Double positive cells were rarely detected in the human kidney. However, in autopsy kidney samples of COVID-19 patients, SARS-CoV-2 nucleoprotein was detected in both ACE2^+^ and TMPRSS2^+^ renal tubular cells, whereas the RNAscope^®^ Assay targeting the SARS-CoV-2 Spike gene was positive mainly in the distal tubular cells and seldom in the proximal tubular cells. In addition, we observed that TMPRSS2 and kidney injury marker protein levels were significantly higher in the SARS-CoV-2-infected renal distal tubular cells, whereas ACE2^+^ proximal tubular cells did not show these changes of kidney injury. These results indicated that the kidney is a direct target for SARS-CoV-2 infection, and SARS-CoV-2-mediated kidney injury mainly occurred in the distal tubular cells. SARS-CoV-2 may enter the renal distal tubular cells without binding to ACE2. Another potential protein may serve as the receptor for SARS-CoV-2 on TMPRSS2^+^ cells. To the best of our knowledge, this is the first report on the precise location of ACE2 and TMPRSS2 in the renal tissue and also the first report of *in situ* RNA analysis showing that the SARS-CoV-2 Spike gene was positive mainly in the distal tubular cells and seldom in the proximal tubular cells. Therefore, this study provides direct evidence of the invasion of SARS-CoV-2 into the kidney tubular cells, and these findings will greatly add to the current understanding of SARS-CoV-2 infection.

Previous studies reported that KIM-1/TIM-1 is upregulated in AKI kidney samples and it is highly expressed in renal damage of various etiologies (Ajay et al., 2014; Tajima et al., 2019). In this study, we found that TIM-1 was mainly upregulated in the distal tubule of all the severe COVID-19 kidney samples, whereas rarely expressed in the proximal tubules. In addition, inflammation-related molecules, which have been proven to be related to AKI in previous studies (Munshi et al., 2011; Tajima et al., 2019; Gabarre et al., 2020), including MCP-1 and IL-6, are also upregulated and enriched in the kidney distal tubules of COVID-19 patients. These results also demonstrated that SARS-CoV-2-mediated kidney injury mainly targeted the distal tubular cells, consistent with our previous analysis.

The renal distal tubule was highly damaged in severe COVID-19 patients. We also investigated whether SARS-CoV-2 could get into the urine. We found that SARS-CoV-2 RNA in urine sediments was positive in 2 out of 15 severe COVID-19 cases. In addition to SARS-CoV-2 being detected in COVID-19 patients’ distal tubular cells, we believe that the SARS-CoV-2 viruses can be secreted through the human urinary system and the urine samples from COVID-19 patients could be infectious as reported by Sun et al. (2020).

AKI is one of the most prevalent complications among patients hospitalized for a wide range of diagnoses (Ajay et al., 2014). The rate of AKI reported among COVID-19 patients has ranged from 0.5 to 36.6%(Chen N. et al., 2020; Chen T. et al., 2020; Cheng et al., 2020a; Diao et al., 2020; Gabarre et al., 2020; Guan et al., 2020; Hirsch et al., 2020; Huang et al., 2020; Wang et al., 2020; Yang et al., 2020; Zhou et al., 2020). Cheng et al. (2020b) reported that AKI occurs in 5.1% of 701 patients from Wuhan, China, whereas in 36.6% of 5,449 patients from New York, United States (Hirsch et al., 2020). We think that the difference was caused by different constituent ratios of critical cases in different cohorts. In addition, a systematic review and meta-analysis of 40 studies and 24,527 patients has found that AKI is associated with severe infection and fatality in patients with COVID-19 (Shao et al., 2020). In the present study, we found that AKI occurred in 9 out of 484 severe patients (1.86%) and 50 out of 190 critical patients (26.3%), but none of the moderate patients had this complication, suggesting that both severe and critical COVID-19 patients were more likely to develop AKI. In fact, 45 (77.6%) out of 58 patients with AKI died at the end. In general, the retrospective cohort study of 722 COVID-19 patients confirmed that AKI was significantly related to more serious disease stages and poor prognosis of COVID-19.

Previous studies have reported many risk factors for generally poor outcomes of COVID-19, including older age (Chen R. et al., 2020; Xu et al., 2020; Yan et al., 2020; Zhou et al., 2020), male sex (Zhang et al., 2020), higher Sequential Organ Failure Assessment (SOFA) score (Zhou et al., 2020), higher body mass index (BMI; Simonnet et al., 2020), and AKI (Cheng et al., 2020a, b; Gabarre et al., 2020; Shao et al., 2020; Xu et al., 2020; Yan et al., 2020). Cox proportional hazard regression confirmed that elevated Scr and BUN, a low estimated glomerular filtration rate (eGFR), and AKI stage 1, stage 2, and stage 3 were independent risk factors for in-hospital COVID-19 patients’ death (Cheng et al., 2020b; Zahid et al., 2020). The pathophysiology of AKI associated with COVID-19 could be related not only to non-specific mechanisms but also to COVID-specific mechanisms, such as direct cell injury caused by SARS-CoV-2 entry through the receptor (ACE2), which is highly expressed in the kidney, imbalance of the renin–angiotensin–aldosterone system, and pro-inflammatory cytokines caused by the SARS-CoV-2 infection and thrombotic events (Gabarre et al., 2020). In our analysis, the median age of the no AKI group was 60 years old, whereas the AKI group was significantly older. Compared with female patients, male patients show higher potential to develop AKI. When comparing laboratory indexes between COVID-19 patients with or without AKI, LDH, BUN, and CRP were associated with increased risk of AKI, in which BUN shows the highest OR (1.12, 95% CI, 1.04–1.20). Subsequently, we investigated the level of these variables of COVID-19 patients on admission, 7 and 14 days after admission, and at the end of the follow-up, respectively. Higher LDH and CRP levels were related to increased risk of AKI during hospitalization. In addition, BUN level showed a continuous increase during hospitalization in AKI patients. Previous studies have shown that elevated BUN is an independent risk factor for death of hospitalized COVID-19 patients (Cheng et al., 2020b), whereas the BUN/Scr ratio had a statistical significance to predict mortality in AKI patients (Yoon et al., 2017), although the change of Scr, a late marker of AKI, is considered only as the diagnostic basis of AKI (Elhmidi et al., 2011; Ugwuowo et al., 2020). However, generally speaking, BUN and Scr have several limitations including poor specificity and sensitivity in monitoring kidney function (Tajima et al., 2019). After kidney injury, BUN and Scr levels may be within the normal range immediately. Our study found that during AKI, BUN showed an upward trend, which indicated that the patient’s condition deteriorated. At this time, it is necessary to comprehensively consider various indicators of patients to evaluate the progress of COVID-19 patients.

In conclusion, these studies provide a direct evidence of the invasion of SARS-CoV-2 into the renal distal tubular cells and lead to AKI. AKI was significantly related to more serious disease stages and poor prognosis of COVID-19 patients. In addition, the progressive increase of BUN during the course of COVID-19 suggests that the patient’s condition is aggravated. At this time, it is necessary to comprehensively evaluate the clinical indicators of the patient to judge the patient’s condition. These results will greatly increase the current understanding of SARS-CoV-2 infection.

## Materials and Methods

### Public Data Acquisition

Three different scRNA-seq datasets of normal kidneys were used in this paper. Two datasets were profiled based on 10X Genomics platform, whereas the other was sequenced with Microwell technology (Young et al., 2018; Han et al., 2020; Liao et al., 2020). Unique molecular identifiers (UMIs) count matrix was constructed by the original authors.

### Quality Control and Combination of scRNA-seq Data

For 10X Genomics-based data, single cells with less than 500 UMIs detected or with over 20% of transcripts derived from the mitochondria were considered as low-quality cells and were filtered out. Subsequently, in order to remove potential doublets, single cells with over 6,000 genes detected per cell were also removed. In order to eliminate potential batch effect between samples and cohorts, sample numbers were used as batch numbers and input into harmony algorithm (Korsunsky et al., 2019). All the downstream analysis was done with Seurat (version 3.0.1) in R environment (version 3.6.1).

For Microwell-based datasets, single cells with less than 200 UMIs detected per cell and over 30% mitochondrial-derived transcripts were removed. Single cells with over 4,000 genes detected were also not included in the downstream analysis. This threshold was determined based on the sequencing depth of different datasets. Subsequently, sample numbers were used as batch numbers to remove potential batch effect in harmony, similar to the 10X Genomics-based datasets.

### Dimension Reduction and Cell Types Identification of scRNA-seq Data

Top 30 harmony corrected dimensions were used in t-SNE plot construction for both datasets. Clustering was conducted with FindCluster function of Seurat. FindAllMarker function was used to identify marker genes of each cell cluster. These machine-learning-based clusters were mapped to known cell types with classical markers provided in previous studies (Lake et al., 2019; Stewart et al., 2019): proximal tubule (LRP2^+^), connecting tubule (CALB1^+^), distal convoluted tubule (SLC12A3^+^), intercalated cell (ATP6V0D1^+^), endothelial cell (CD39^+^), myeloid cell (LYZ^+^), collecting duct principal cell (AQP2^+^), fibroblast (DCN^+^), plasma cell (CD79^+^JCHAIN^high^), T cell (CD3D^+^), LOH (SLC12A1^+^), erythrocyte (HBB^+^), mast cell (TPSAB1^+^), podocyte (NHPS2^+^), and urothelial epithelial (KRT13^+^).

### Kidney Tissue Sample Collection

Kidney samples were obtained from autopsies of 10 COVID-19 cases (February 18 to March 27, 2020). Tissue specimens were fixed in 2.5% glutaraldehyde for 36 h before the following procedures.

### IFC Staining Assay

Severe acute respiratory syndrome coronavirus 2 nucleoprotein was co-stained with ACE2 and TMPRSS2, respectively. Anti-SARS-CoV-2 nucleoprotein antibody (Cat No. 40143-T62; Sino Biological), anti-TMPRSS2 antibody (ab109131; Abcam), and anti-ACE2 antibody (ab108252; Abcam) were used in this assay.

### Localization of SARS-CoV-2 RNA in the Kidney Tissues

A novel *in situ* RNA analysis platform for formalin-fixed, paraffin-embedded tissues, RNAscope^®^ Assay (Wang et al., 2012), was performed to detect SARS-CoV-2 RNA in COVID-19 patients’ kidney specimens using a SARS-CoV-2-specific probe (Advanced Cell Diagnostics, Inc., United States), following the manufacturer’s protocol. This probe (v-nCoV2019-S) was used to target the SARS-CoV-2 Spike gene.

### Histological Evaluation and IHC Staining Assay

Tissue specimens were fixed with 2.5% glutaraldehyde and embedded in paraffin wax. Serial sections (5 μm thick) were prepared for H&E staining and IHC staining. Furthermore, the prepared slides were scanned as high-resolution digital images using the Pannoramic MIDI II (3DHistech, Budapest, Hungary) histological scanner. The following antibodies were used in IHC staining assay: anti-TMPRSS2 antibody (ab109131; Abcam), anti-ACE2 antibody (ab108252; Abcam), anti-TIM-1 antibody (ab47635; Abcam), anti-IL-6 antibody (ab6672; Abcam), and anti-MCP-1 antibody (ab9669; Abcam). The immunoreactive score (IRS) gives a range of 0–12 as a product of multiplication between positive cells proportion score (0–4) and staining intensity score (0–3). The IRS (0–1, negative; 2–3, mild; 4–8, moderate; 9–12, strong positive) measured the expression of ACE2, TMPRSS2, TIM1, IL-6, and MCP-1.

Because there is no unified marker of the proximal tubule and distal tubule at present, in this study, we identified it by traditional H&E staining. Identification on the basis of: the epithelial cells of the proximal tubules are cubic or tapered, with unclear cell boundaries, large cell bodies, eosinophilic cytoplasm, round nuclei, and brush border on the lumen of the epithelial cells. The lumen of the distal tubule is large and regular, the epithelial cells are cuboid, smaller than the proximal tubule cells, the nucleus is located in the center or near the lumen, the cytoplasm staining is shallower than the proximal tubule, and the lumen of the epithelial cells has no brush border. During this study, the identification of the proximal tubules and distal tubules was done by a pathologist through H&E staining.

### SARS-CoV-2 RNA Detection in Urine Samples

Coronavirus disease 2019 patient urine specimens were obtained and stored between 2 and 8°C until detection of SARS−CoV−2 RNA. Briefly, SARS-CoV-2 RNA was isolated from a 600 μl sample using a Nucleic Acid Extraction and Purification Kit (SUPI-1017; Supbio, Guangzhou, China), according to the manufacturer’s protocol. The levels of SARS-CoV-2 RNA were detected by quantitative real-time polymerase chain reaction (qRT-PCR) using SARS-CoV-2 RNA Real-Time PCR Kit (Suzhou Bacme Biotech Co., Ltd.).

### Clinical Information Collection

Seven hundred twenty-two COVID-19 patients treated at the West Branch of Union Hospital of Huazhong University of Science and Technology in Wuhan, China from January 26 2020 to March 31, 2020 were involved in this study. Demographic, clinical, treatment, laboratory, and outcome data were obtained from electronic medical records. All data were counted by two physicians and checked by another one to adjust potential mistakes.

This study was conducted in accordance with the principles of the Declaration of Helsinki and approved by the Medical Ethical Committee of Tongji Medical School, Huazhong University of Science and Technology (S100). According to the Diagnosis and Treatment Protocol for Novel Coronavirus Pneumonia (Trial Version 7) published by the General Office of China Health Commission (Wei, 2020), all the patients were diagnosed with COVID-19 and classified into moderate, severe, and critical types.

Moderate cases (Wei, 2020): showing fever and respiratory symptoms with radiological findings of pneumonia.

Severe cases (Wei, 2020): adult cases meeting any of the following criteria: (1) respiratory distress (≥30 breaths/min); (2) oxygen saturation ≤93% at rest; and (3) arterial partial pressure of oxygen (PaO_2_)/fraction of inspired oxygen (FiO_2_) ≤300 mmHg (1 mmHg = 0.133 kPa). Cases with chest imaging that shows obvious lesion progression within 24–48 h >50% shall be managed as severe cases. Child cases meeting any of the following criteria: (1) tachypnea [respiratory rate (RR) ≥ 60 breaths/min for infants aged below 2 months, RR ≥ 50 BPM for infants aged 2–12 months, RR ≥ 40 BPM for children aged 1–5 years, and RR ≥ 30 BPM for children above 5 years old] independent of fever and crying; (2) oxygen saturation ≤92% on finger pulse oximeter taken at rest; (3) labored breathing (moaning, nasal fluttering, and infrasternal, supraclavicular, and intercostal retraction), cyanosis, and intermittent apnea; (4) lethargy and convulsion; and (5) difficulty feeding and signs of dehydration.

Critical cases, cases meeting any of the following criteria (Wei, 2020): (1) respiratory failure and requiring mechanical ventilation; (2) shock; and (3) with other organ failure that requires ICU care.

### Statistical Analysis

Area under the curve score of ROC analysis was calculated in R studio with pROC package (Robin et al., 2011). Categorical variables were listed as frequencies or percentages and analyzed with Chi-square test or Fisher’s exact test. Continuous variables were presented as median (IQR). The Mann–Whitney *U* test or Kruskal–Wallis test was used to compare continuous variables. Multi-comparison between groups was performed with Bonferroni adjustment method. In order to construct a more accurate model, variables with AUC > 0.6 and show statistical significance in univariate logistic regression were input into multivariable logistic regression model. Stepwise method was used to optimize the model. Hosmer and Lemeshow test and ROC analysis were conducted to confirm the goodness of fit of this multivariable model. In the multi-time point analysis, patients with missing value were excluded. All the analysis was finished in R studio (version 1.2.5033). A two-sided *p*-value < 0.05 was considered statistically significant.

## Data Availability Statement

The original contributions presented in the study are included in the article/Supplementary Material, further inquiries can be directed to the corresponding authors.

## Ethics Statement

The studies involving human participants were reviewed and approved by the Medical Ethical Committee of Tongji Medical School, Huazhong University of Science and Technology. Written informed consent to participate in this study was provided by the participants’ legal guardian/next of kin.

## Author Contributions

Y-MW, KC, and LLa designed the study and helped to revise the manuscript. ZC, JH, and LLl wrote the manuscript. RC, MW, MX, Z-QL, YZ, HL, CG, and JZ extracted the clinically relevant data. JH, LLl, and MX performed the experiment. ZC, JH, and LLl analyzed the data. All authors read and approved the final manuscript.

## Conflict of Interest

The authors declare that the research was conducted in the absence of any commercial or financial relationships that could be construed as a potential conflict of interest.
