# Supplementary material for: SARS-CoV-2 Causes Acute Kidney Injury by Directly Infecting Renal Tubules
Source: Front Cell Dev Biol. 2021 May 31;9:664868. doi: 10.3389/fcell.2021.664868 (PMC8201778; doi:10.3389/fcell.2021.664868)

Supplementary Figures and legends

Supplementary Figure 1. The expression of ACE2 and TMPRSS2 in different human kidney cells. (A) Proportion of different cells in ACE2- TMPRSS2-, ACE2+, ACE2+ TMPRSS2+, TMPRSS2+ cell population. (B) The number of different cells in ACE2- TMPRSS2-, ACE2+, ACE2+ TMPRSS2+, TMPRSS2+ cell population. IC, intercalated cell; EC, endothelial cell; MC, myeloid cell; DT, distal tubule; UE, urothelial epithelial; PT, proximal tubule; CD, collecting duct; DCT, distal convoluted tubule; CNT, connecting duct; SMC, smooth muscle cell.

Supplementary Figure 2. SARS-CoV-2 nucleoprotein was detected in kidney tissues. (A-B) SARS-CoV-2 nucleoprotein was co-stained with ACE2 or TMPRSS2 by Immunofluorescence (IFC) staining in normal kidney samples (n=3).

Supplementary Figure 3. The results of the effectiveness analysis of the antibodies involved in IHC.

Supplementary Figure. 1

A

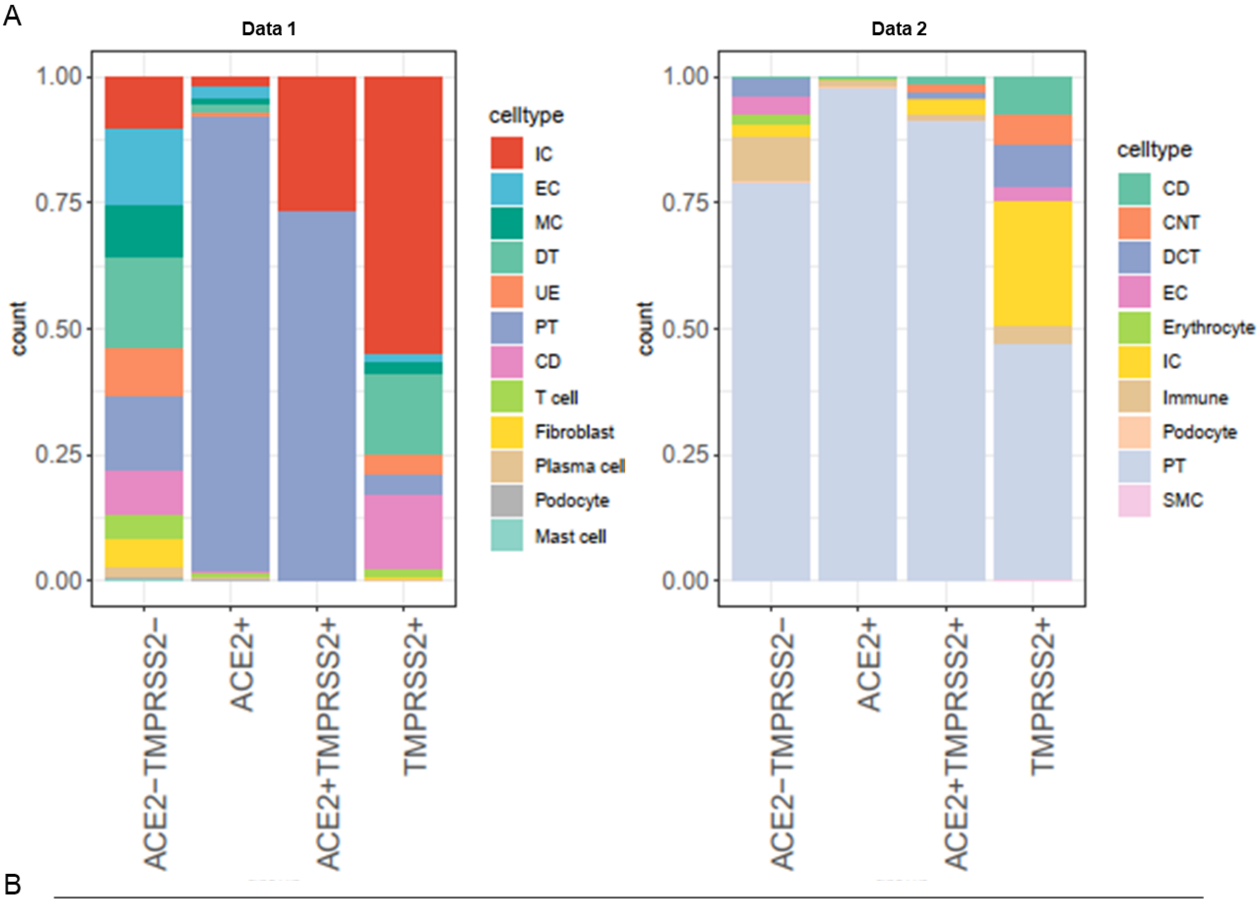

B

Data 1	IC	EC	MC	DT	UE	PT	CD	T cell	Fibroblast	Plasma cell	Podocyte	Mast cell
ACE2- TMPRSS2-	2539	3873	2708	4509	2480	3677	2252	1306	1399	452	143	82
ACE2+	12	14	8	11	3	565	2	4	2	2	1	1
ACE2+ TMPRSS2+	5	0	0	0	0	14	0	0	0	0	0	0
TMPRSS2+	848	29	34	246	66	61	223	27	8	4	1	1

Data 2	CD	CNT	DCT	EC	Erythrocyte	IC	Immune	Podocyte	PT	SMC
ACE2- TMPRSS2-	112	107	1576	1552	1014	1058	3935	42	35675	77
ACE2+	2	1	7	6	6	8	38	2	3318	0
ACE2+ TMPRSS2+	1	1	1	0	0	2	1	0	62	0
TMPRSS2+	49	39	53	19	1	161	22	0	306	2

Supplementary Figure. 2

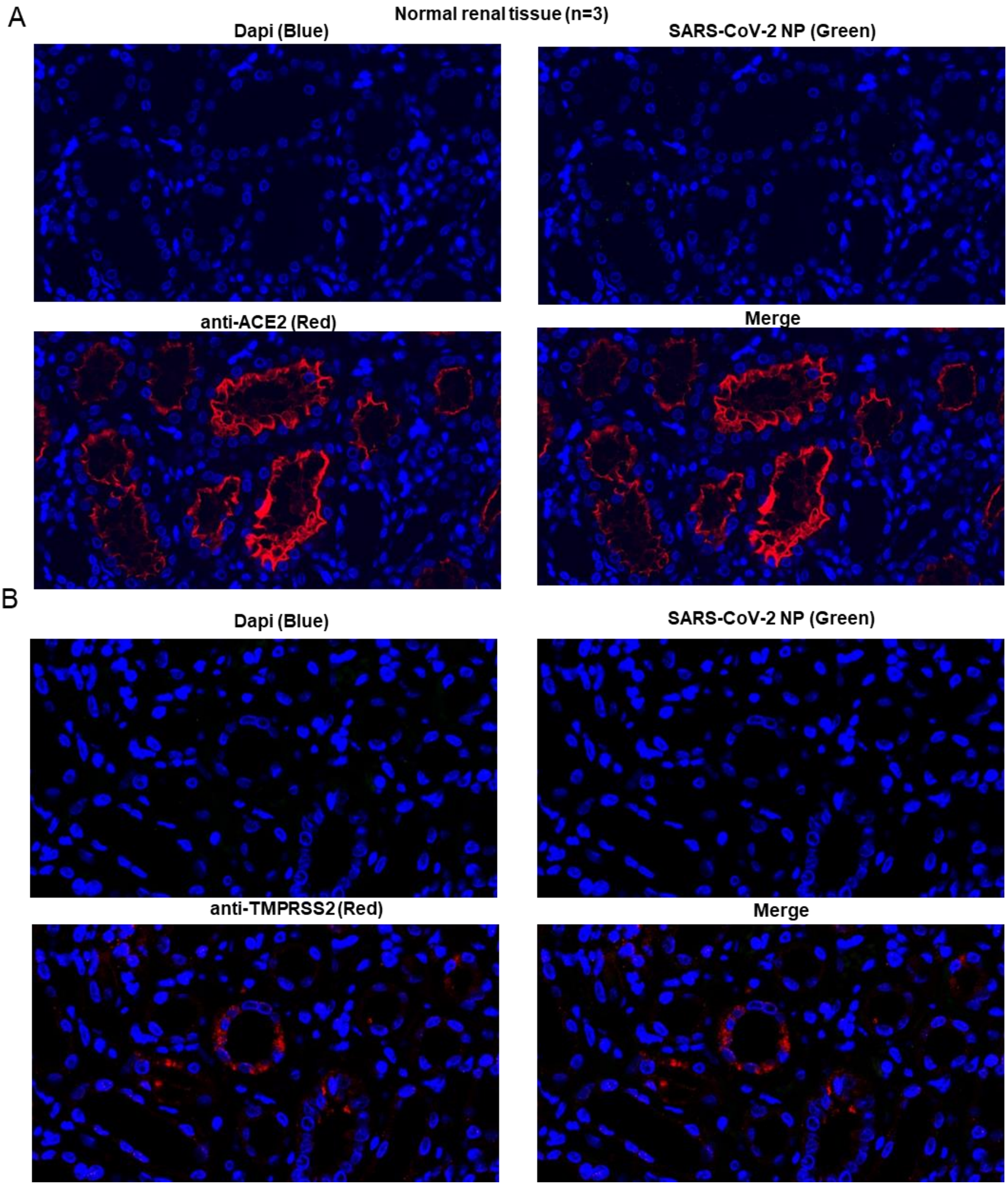

Supplementary Figure. 3

Normal renal tissue (n=3)

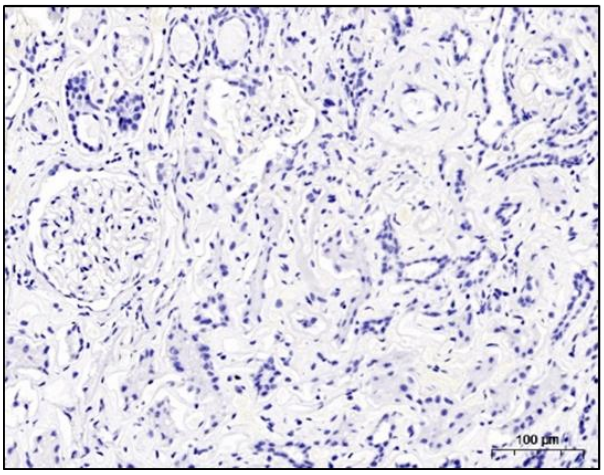

COVID-19 renal tissues (n=10)

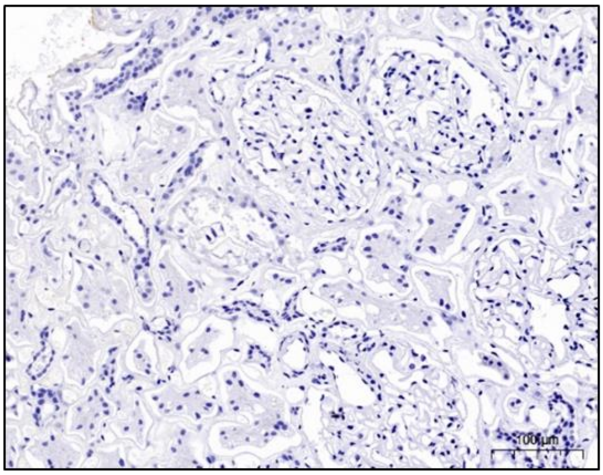

Supplement: Supplementary file 1 [file Data_Sheet_1.PDF]
